# Diagnostic accuracy of a point-of-care urine test for tuberculosis screening among newly-diagnosed hiv-infected adults: a prospective, clinic-based study

**DOI:** 10.1186/1471-2334-14-110

**Published:** 2014-02-26

**Authors:** Paul K Drain, Elena Losina, Sharon M Coleman, Janet Giddy, Douglas Ross, Jeffrey N Katz, Rochelle P Walensky, Kenneth A Freedberg, Ingrid V Bassett

**Affiliations:** 1 Division of Infectious Diseases and Medical Practice Evaluation Center, Massachusetts General Hospital, Boston, USA; 2 Brigham and Women’s Hospital, Boston, USA; 3 Boston University School of Public Health, Boston, USA; 4 McCord Hospital, Durban, South Africa; 5 St. Mary’s Hospital, Durban, South Africa; 6 Medical Practice Evaluation Center, Department of Medicine, Massachusetts General Hospital, 50 Staniford St, #943, Boston, MA 02114, USA

**Keywords:** Tuberculosis, HIV/AIDS, Lipoarabinomannan (LAM), Urine, Diagnostic testing, Screening, South Africa

## Abstract

**Background:**

A rapid diagnostic test for active tuberculosis (TB) at the clinical point-of-care could expedite case detection and accelerate TB treatment initiation. We assessed the diagnostic accuracy of a rapid urine lipoarabinomannan (LAM) test for TB screening among HIV-infected adults in a TB-endemic setting.

**Methods:**

We prospectively enrolled newly-diagnosed HIV-infected adults (≥18 years) at 4 outpatient clinics in Durban from Oct 2011-May 2012, excluding those on TB therapy. A physician evaluated all participants and offered CD4 cell count testing. Trained study nurses collected a sputum sample for acid-fast bacilli smear microscopy (AFB) and mycobacterial culture, and performed urine LAM testing using Determine™ TB LAM in the clinic. The presence of a band regardless of intensity on the urine LAM test was considered positive. We defined as the gold standard for active pulmonary TB a positive sputum culture for *Mycobacterium tuberculosis*. Diagnostic accuracy of urine LAM was assessed, alone and in combination with smear microscopy, and stratified by CD4 cell count.

**Results:**

Among 342 newly-diagnosed HIV-infected participants, 190 (56%) were male, mean age was 35.6 years, and median CD4 was 182/mm^3^. Sixty participants had culture-positive pulmonary TB, resulting in an estimated prevalence of 17.5% (95% CI 13.7-22.0%). Forty-five (13.2%) participants were urine LAM positive. Mean time from urine specimen collection to LAM test result was 40 minutes (95% CI 34–46 minutes). Urine LAM test sensitivity was 28.3% (95% CI 17.5-41.4) overall, and 37.5% (95% CI 21.1-56.3) for those with CD4 count <100/mm^3^, while specificity was 90.1% (95% CI 86.0-93.3) overall, and 86.9% (95% CI 75.8-94.2) for those with CD4 < 100/mm^3^. When combined with sputum AFB (either test positive), sensitivity increased to 38.3% (95% CI 26.0-51.8), but specificity decreased to 85.8% (95% CI 81.1-89.7).

**Conclusions:**

In this prospective, clinic-based study with trained nurses, a rapid urine LAM test had low sensitivity for TB screening among newly-diagnosed HIV-infected adults, but improved sensitivity when combined with sputum smear microscopy.

## Background

*Mycobacterium tuberculosis* accounts for 25% of the 2 million AIDS-related deaths annually, and global health leaders have set a target of detecting the vast majority of infectious tuberculosis cases by 2015 [1,2]. However, given the existing diagnostic tests, implementing a sensitive, cost-effective screening strategy among HIV-infected adults will be challenging [3]. Sputum smear microscopy has poor diagnostic sensitivity among HIV-infected South African adults with culture-confirmed pulmonary tuberculosis [4-6]. Mycobacterial sputum culture, the *de facto* gold standard diagnostic test, is not widely available in resource-limited settings due to its time- and labor–intensive nature, and is only available at 53% of health facilities in sub-Saharan Africa [7,8]. The Xpert MTB/RIF assay is a substantial advancement in tuberculosis diagnostics, but the assay’s high costs, operator time, and reliance on electricity may render it impractical at the clinical point-of-care in many settings [9,10]. The World Health Organization’s Stop TB Department still identifies research and development of novel diagnostics as a research priority [11,12].

Lipoarabinomannan (LAM), a lipopolysaccharide that forms a major component of the cell wall of tuberculosis, is released from metabolically active or degrading organisms, and is filtered by the kidneys and excreted in urine [13]. A laboratory-based urine LAM ELISA test had varying levels of test sensitivity, but high specificity, for detecting active tuberculosis [4,14-17]. A lateral flow assay for urinary LAM (Determine™ TB LAM; Alere, Waltham, USA) was developed to return results in less than 30 minutes and without reliance on laboratory equipment or reagents. We sought to prospectively assess the diagnostic accuracy of the rapid urine LAM test when used by trained nurses in a clinical-setting for tuberculosis screening among newly-diagnosed HIV-infected adults.

## Methods

### Study sites and participants

We conducted a prospective clinic-based study of newly-diagnosed HIV-infected adults who presented for voluntary HIV counseling and testing from October 2010 to May 2011. Enrollment, specimen collection, and urine LAM testing were conducted in outpatient clinical areas of McCord Hospital, St. Mary’s Hospital, and two municipal health clinics in KwaZulu-Natal, South Africa. McCord Hospital is an urban, state-aided general hospital that serves the greater Durban area. St. Mary’s Hospital in Mariannhill is a state-aided general hospital that serves a resource-limited population in a peri-urban area of Durban. Both McCord Hospital and St. Mary’s Hospital operate high-volume outpatient HIV clinics that had been providing ART since 2001 and 2003, respectively, and receiving President’s Emergency Plan for AIDS Relief (PEPFAR) support since 2004. The two municipal primary health clinics, Tshelimnyama and Mariannridge, are primary health care clinics located within the catchment area of St. Mary’s Hospital. Throughout the course of the study, all four clinical sites offered free HIV counseling and rapid testing during normal business hours.

We offered enrollment to adults (≥18 years) newly diagnosed with HIV on the same day. We excluded those already known to be HIV-infected, pregnant, or unwilling to share their HIV test results with the research team. All participants provided written informed consent either in English or Zulu. The ethics committees of McCord Hospital [IRB00005803] and St. Mary’s Hospital in Durban, and Partners HealthCare in Boston [Protocol #: 2006-P-001379/40] approved the study.

### Data collection

Prior to study commencement, a representative from Alere Inc. conducted a training session for 3 study nurses, each of whom had previously received training in the diagnosis, treatment, and care of tuberculosis-infected patients. The training session reviewed the function, procedure, and interpretation of the Determine™ TB LAM test (Alere Inc., Waltham, USA). Each nurse practiced performing and interpreting the urine LAM test with direct supervision from the representative, and became comfortable conducting clinic-based urine LAM testing using the provided reference scale card.

Upon enrollment, study nurses collected demographic details, self-reported history of prior tuberculosis care and treatment, signs and symptoms of tuberculosis using a standardized questionnaire, and current use of diuretic medications that may alter urine LAM results [18]. Study nurses obtained a respiratory sputum sample and a urine specimen in a sterile container. Participants unable to provide an expectorated respiratory sputum sample received sputum induction with nebulized 3% hypertonic saline using a portable machine (WH-802, Yuehua Medical Instrument Factory Co.; Guangdong, China).

Study nurses performed urine LAM testing in accordance with the manufacturer’s specifications at the clinical point-of-care. LAM test results were not used to guide therapeutic decisions. All LAM tests used were from Manufacturer Lot Numbers 110512, 120215, or 120222. The LAM test kits were maintained in a sealed pouch at room temperature (15 – 26°C), and always within the manufacturer’s required range of 2 – 30°C. All urine samples were tested immediately after urine collection. If there was an interruption in LAM test supply and the sample could not be tested within 8 hours of specimen collection, urine samples were stored in a −20°C frost-free freezer (actual temperature ranged from −18° to −22°C). When frozen urine samples were tested, each specimen was brought to room temperature for at least one hour prior to testing, and thawed samples were centrifuged at 10,000 G for 5 minutes at room temperature. For all tests, 60 microliters of urine or clear supernatant was pipetted to the LAM test strip, and the nurse from the respective clinic of the participant interpreted test results.

Due to prior concerns of poor test sensitivity, each nurse conducted two urine LAM tests on one urine sample from each participant. One nurse at each site interpreted test results within 25–35 minutes, and recorded the time to test result based on a portable timer, under ambient lighting conditions. Participants were considered urine LAM positive if either urine LAM test was positive by the appearance of a color band, as defined by the manufacturer, and tests were not scored. Nurses had access to a small reference card supplied by the manufacturer. If either urine LAM test result was not interpretable, the study nurse repeated the test using the same urine sample. The urine samples were then labeled and transported to a laboratory at the University of KwaZulu-Natal for storage in a -20C freezer.

Respiratory sputum samples were transported to a reference laboratory at the University of KwaZulu-Natal for smear microscopy (AFB) and mycobacterial culture. Certified technologists at a reference laboratory performed smear microscopy using both Ziehl-Neelsen and Auramine stains. Before staining for AFB, sputum samples were decontaminated with N-acetyl L Cysteine and NaOH to a final concentration of 1.25% before being centrifuged at 3,000 revs for 20 minutes and resuspended in 1 ml of 7H9 broth. Sputum samples were inoculated onto Middlebrook 7H11 solid agar medium and 0.5 ml of sample were used for liquid mycobacterial growth indicator (MGIT) tubes (automated Bactec 960 instrument). Culture plates were read at 3 and 6 weeks. We identified positive MGIT or solid agar cultures as *Mycobacterium tuberculosis* using niacin and nitrate testing, and all isolates from positive cultures underwent drug susceptibility testing.

All participants were evaluated by a non-study clinician as part of their usual care, offered CD4 count testing, and received additional diagnostic evaluations, such as a chest X-ray or abdominal ultrasound, when indicated. All HIV testing, care, and treatment were provided in accordance with current South African Department of Health HIV testing and treatment guidelines [19]. All participants were followed for 9 months for assessment of anti-tubercular therapy initiation.

### Statistical analyses

We analyzed and reported the study results in accordance with the STAndards for Reporting of Diagnostic accuracy studies (STARD) [20]. We excluded participants taking anti-tubercular therapy at the time of LAM testing, as well as those unable to produce a sputum or urine sample, from all analyses. The AFB was recorded as positive if either the Ziehl-Neelsen or Auramine stain was positive. We compared urine LAM results to “culture-confirmed pulmonary tuberculosis”, which we defined as positive sputum culture for *M. tuberculosis*, the accepted gold standard. Because mycobacterial culture is an imperfect gold standard test for active tuberculosis [21], we also compared urine LAM results to “clinically suspected tuberculosis”, which we defined as having either culture-confirmed pulmonary tuberculosis, a diagnosis of extrapulmonary tuberculosis, or having been initiated on anti-tubercular therapy by a clinician.

The study population was characterized with simple descriptive statistics. We calculated LAM test sensitivity, specificity, likelihood ratios (positive and negative), and predictive values for urine LAM and sputum AFB for sputum culture-confirmed pulmonary tuberculosis. We repeated the same analyses when using a combined screening strategy with both urine LAM and sputum AFB testing. For this combined analysis, a screening test was considered positive if either the urine LAM or sputum AFB results were positive. We conducted overall analysis and also assessed LAM test accuracy characteristics, stratified by CD4 cell count, and calculated exact 95% confidence intervals (CI) for estimates of prevalence and diagnostic accuracy. We also evaluated the diagnostic test performance characteristics of urine LAM using the secondary outcome, “clinically suspected tuberculosis”. We did not assess diagnostic accuracy of sputum AFB for “clinically suspected tuberculosis”, since AFB test result was used to guide anti-tubercular therapy initiation. We assessed whether the primary or the secondary case definition of tuberculosis were associated with gender, prior tuberculosis infection, or diuretic use. Sensitivity and specificity were defined as the true positive and true negative rates, respectively. Likelihood ratio positive (and negative) was the probability of a person who has the disease testing positive (negative) divided by the probability of a person who does not have the disease testing positive (negative). We calculated the change in clinical pre-test probability, by multiplying the pre-test odds by the positive or negative likelihood ratio and then converting back to a post-test probability, when using a combined screening strategy with both urine LAM and sputum AFB testing. All reported p-values were two-tailed, and a p-value <0.05 was considered statistically significant. We conducted analyses using SAS software (version 9.2; SAS Institute, Cary, NC).

### Role of the funding source

The sponsor of the study had no role in study design, data collection, data analysis, data interpretation or writing of the manuscript. The corresponding and senior authors had full access to all the data in the study and had final responsibility for the decision to submit for publication.

## Results

**Figure 1:**
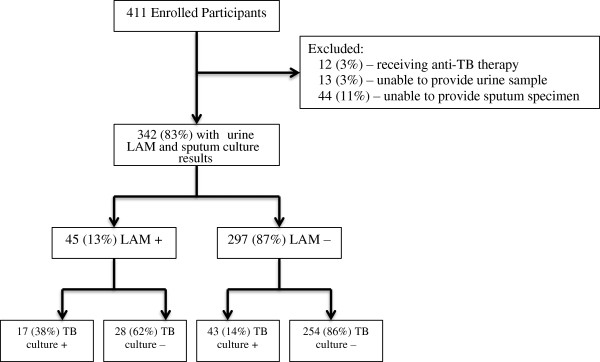
Participant flow diagram.

Among the 411 newly-diagnosed HIV-infected adults enrolled, 342 (83.2%) participants completed urine LAM and sputum culture testing, and were not receiving anti-tubercular therapy (Figure 1). Forty-five (13.2%) were urine LAM positive, with an estimated population prevalence of 9.8-17.0%. Based on this sample, the estimated sputum culture-positive tuberculosis prevalence was 17.5% (95% CI 13.7-22.0%). The mean age was 35.6 years (S.D. ±9.8 years), and 190 (55.6%) participants were male (Table 1). Twenty-nine (8.5%) participants reported a history of previous tuberculosis disease. Three (0.9%) participants were taking diuretic medications at the time of LAM testing. Two hundred-eighty (81.9%) underwent CD4 testing; the median CD4 cell count was 182/mm^3^ (IQR 70-298/mm^3^). Among the entire cohort, 44 people (11%) were unable to provide an expectorated or induced sputum sample, while 13 people (3%) were unable to provide a urine sample.

**Table 1:** Characteristics of HIV-infected participants (N = 342)

	**Mean ± S.D. or N (%)**
*Demographics*	
Age (years)	35.6 ± 9.8
Male gender	190 (55.6)
*Tuberculosis history*	
Received treatment for prior TB	26 (7.6)
*Current diuretic use*	
Yes	3 (0.9)
No	336 (98.2)
Don’t know	3 (0.9)
*CD4 cell count (median/IQR)/mm*^3*^	182 (70–298)
≥350 cells/mm^3^	58 (20.7)
200-349 cells/mm^3^	74 (26.4)
100-199 cells/mm^3^	55 (19.6)
<100 cells/mm^3^	93 (33.2)
*Tuberculosis testing*	
Sputum smear microscopy (AFB) positive	24 (7.0)
Urine LAM positive	45 (13.2)
Sputum AFB or urine LAM positive	63 (18.4)
*Tuberculosis outcomes*	
Culture-confirmed pulmonary TB	60 (17.5)
Clinically suspected TB	92 (26.9)

*N = 280.

Among this cohort, 24 (7.0%) participants were sputum smear microscopy (AFB) positive, with an estimated population prevalence of 4.6-10.3%. Sixty-three (18.4%) participants had either a positive sputum AFB or urine LAM test, with an estimated population prevalence of 14.5-22.9% (Table 1). Among urine LAM positive participants, 36 (80.0%) had two positive urine LAM tests, and 9 (20.0%) had one positive urine LAM test. We performed urine LAM testing on the same day as specimen collection for 294 (86.0%) participants. We performed urine LAM testing on frozen samples for 48 (14.0%) participants, as a result of an interruption in LAM test supply. Among the samples tested in the clinic, the mean time from urine specimen collection to obtaining a LAM test result was 40.0 minutes (95% CI 34.2-45.5 minutes).

### Diagnostic accuracy of LAM for culture-confirmed pulmonary tuberculosis

Urine LAM test estimated sensitivity and specificity for diagnosing culture-confirmed pulmonary tuberculosis were 28.3% (95% CI 17.5-41.4%) and 90.1% (95% CI 86.0-93.3%), respectively (Table 2). Urine LAM test sensitivity was 37.5% (95% CI 21.1-56.3%) for those with CD4 cell count <100/mm^3^, which did not differ significantly from those with a CD4 count > l00/mm^3^ (sensitivity =25.0%, 95% CI 9.0-49.0%; p = 0.38). In addition, a test for trend did not find significantly greater sensitivity at lower CD4 count strata (p = 0.46). Estimated test sensitivity and specificity for sputum AFB were 18.3% (95% CI 9.5-30.4%) and 95.4% (95% CI 92.3-97.5%), respectively. The urine LAM test identified 10% more cases of culture-positive pulmonary tuberculosis than sputum AFB. Likelihood ratio (LR) positive and LR negative values for urinary LAM testing were 2.85 (95% CI - 1.67-4.87) and 0.80 (95% CI - 0.68-0.94), respectively. The LR positive and negative for sputum AFB were 3.98 (95% CI 1.87-8.44) and 0.86 (95% CI 0.76-0.97), respectively. Among the 9 LAM discordant results, 2 people were culture-positive for tuberculosis and 1 additional person was clinically suspected of having active tuberculosis. There were no significant differences in baseline characteristics or urine LAM test results among the 48 frozen urine samples compared to those with a fresh urine sample, or participants without a CD4 result compared to those with a CD4 result.

**Table 2:** Diagnostic accuracy of urine LAM and/or sputum smear microscopy (AFB) testing for culture-confirmed pulmonary tuberculosis

	**Sensitivity**		**Specificity**		**LR Positive**	**LR Negative**	**Positive PV**	**Negative PV**
	**N**	**% (95% CI)**	**N**	**% (95% CI)**	**(95% CI)**	**(95% CI)**	**% (95% CI)**	**% (95% CI)**
**Urine LAM alone**								
All participants	17/60	28.3 (17.5-41.4)	254/282	90.1 (86.0-93.3)	2.85 (1.67-4.87)	0.80 (0.68-0.94)	37.8 (23.8-53.5)	85.5 (81.0-89.3)
CD4 ≥ 100 cells/mm^3^	5/20	25.0 (9.0-49.0)	153/167	91.6 (86.3-95.3)	2.98 (1.20-7.41)	0.82 (0.63-1.06)	26.3 (9.0-51.2)	91.1 (85.7-94.9)
CD4 < 100 cells/mm^3^	12/32	37.5 (21.1-56.3)	53/61	86.9 (75.8-94.2)	2.86 (1.30-6.27)	0.72 (0.54-0.96)	60.0 (36.1-80.9)	72.6 (60.9-82.4)
**Sputum AFB alone**								
All participants	11/60	18.3 (9.5-30.4)	269/282	95.4 (92.3-97.5)	3.98 (1.87-8.44)	0.86 (0.76-0.97)	45.8 (25.6-67.2)	85.6 (80.2-88.4)
CD4 ≥ 100 cells/mm^3^	4/20	20.0 (5.7-43.7)	158/167	94.6 (90.0-97.5)	3.68 (1.25-10.89)	0.85 (0.68-1.06)	30.8 (9.0-61.4)	90.8 (85.4-94.6)
CD4 < 100 cells/mm^3^	4/32	12.5 (3.5-29.0)	60/61	98.4 (91.2-99.9)	7.63 (0.89-65.40)	0.89 (0.78-1.02)	80.0 (28.4-99.5)	68.2 (57.4-77.7)
Urine LAM or sputum AFB*								
All participants	23/60	38.3 (26.0-51.8)	242/282	85.8 (81.1-89.7)	2.70 (1.76-4.16)	0.72 (0.59-0.88)	36.5 (24.7-49.6)	86.7 (82.2-90.5)
CD4 ≥ 100 cells/mm^3^	7/20	35.0 (15.4-59.2)	145/167	86.8 (80.6-91.5)	2.64 (1.29-5.39)	0.75 (0.54-1.04)	24.1 (10.3-43.5)	91.7 (86.3-95.5)
CD4 < 100 cells/mm^3^	13/32	40.6 (23.7-59.4)	52/61	85.3 (73.8-93.0)	2.75 (1.32-5.74)	0.70 (0.51-0.94)	59.1 (36.4-79.3)	73.2 (61.4-83.1)

*Test considered positive if either Urine LAM or sputum AFB were positive.

LR = likelihood ratio; PV = predictive value; CI = confidence interval.

When using a combined screening strategy of either urine LAM or sputum AFB positive, test sensitivity and specificity were 38.3% (95% CI 26.0-51.8%) and 85.8% (95% CI 81.1-89.7%). The addition of urine LAM to sputum AFB testing detected an additional 20% of culture-positive pulmonary tuberculosis cases, which was a statistically significant improvement (p = 0.02). With this combined screening strategy, the LR positive and negative values were 2.70 (95% CI 1.76-4.16) and 0.72 (95% CI 0.59-0.88).

### Diagnostic accuracy of LAM for clinically suspected tuberculosis

Within our cohort, 92 (26.9%) participants met our definition of “clinically suspected tuberculosis”, which gives an estimated population prevalence of 22.3-31.9%. Nine (2.6%) participants were diagnosed with extrapulmonary TB, which gives an estimated population prevalence of 1.2-4.9%. Among those 32 participants started on therapy with a negative sputum culture, 9 (28.1%) had positive AFB testing, 6 (18.8%) were diagnosed with extrapulmonary tuberculosis, and 16 (50.0%) were diagnosed based on clinical signs, symptoms, and/or chest radiography. In addition, 1 (3.1%) participant was diagnosed as sputum culture-positive on a sputum sample sent through the hospital’s clinical laboratory, and was started on anti-tubercular therapy, but the sputum culture at our reference laboratory remained negative.

We assessed diagnostic performance characteristics of the urine LAM test using our secondary case definition of clinically-suspected active tuberculosis (Table 3). In these analyses, urine LAM test sensitivity and specificity were 25.0% (95% CI - 16.6-35.1%) and 91.2% (95% CI - 87.0-94.4%), respectively. The LR positive and negative were 2.84 (95% CI - 1.67-4.48) and 0.82 (95% CI - 0.73-0.93). These results were almost identical to the culture-confirmed pulmonary tuberculosis, and CD4 strata had little impact on test characteristics. In addition, diagnostic accuracy of the urine LAM test was not associated with gender, prior tuberculosis infection, or diuretic use for both culture-confirmed and clinical suspected tuberculosis.

**Table 3:** Diagnostic accuracy of urine LAM testing for clinically suspected active tuberculosis

	**N**	**Sensitivity (95% CI)**	**Specificity (95% CI)**	**LR positive (95% CI)**	**LR negative (95% CI)**	**Positive PV (95% CI)**	**Negative PV (95% CI)**
**Urine LAM**							
All participants	92	25.0% (16.6-35.1)	91.2% (87.0-94.4)	2.84 (1.67-4.84)	0.82 (0.73-0.93)	51.1% (35.8-66.3)	76.8% (71.5-81.5)
CD4 ≥ 100 cells/mm^3^	35	22.8% (10.4-40.1)	92.7% (87.3-96.3)	3.14 (1.36-7.22)	0.83 (0.69-1.00)	42.1% (20.3-66.5)	83.8% (77.4-89.1)
CD4 < 100 cells/mm^3^	47	27.7% (15.6-42.6)	84.8% (71.1-93.7)	1.82 (0.80-4.14)	0.85 (0.69-1.06)	65.0% (40.8-84.6)	53.4% (41.4-65.2)

LR = likelihood ratio; PV = predictive value; CI = confidence interval.

### Comparison between sputum AFB and urine LAM testing

**Figure 2:**
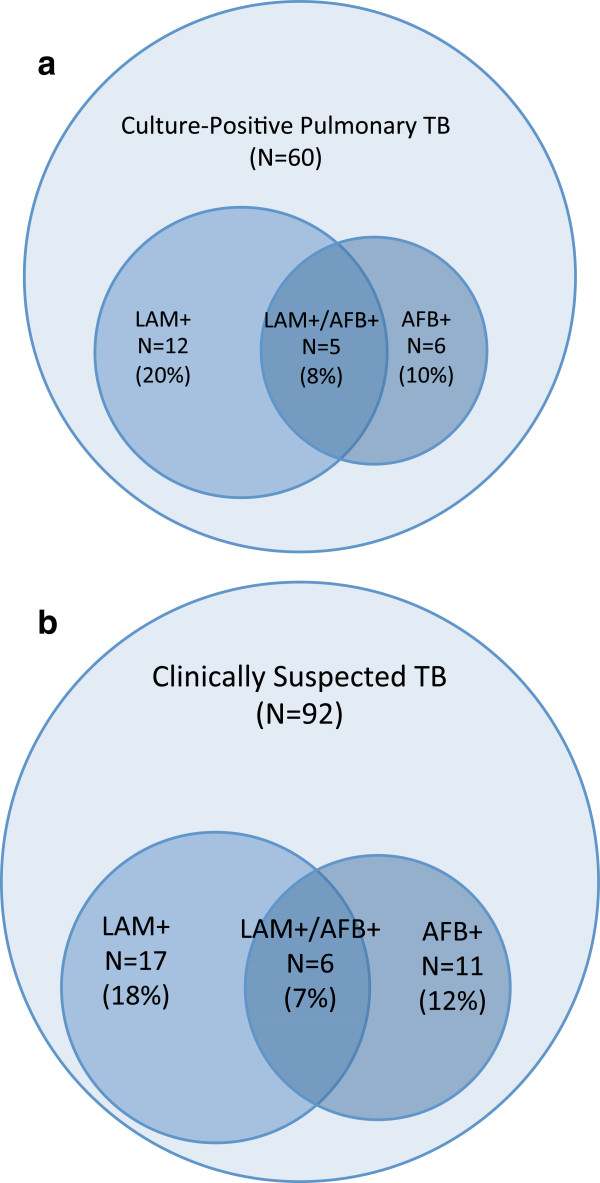
**Urine LAM and sputum smear microscopy (AFB) to diagnose tuberculosis among those who were either culture-confirmed (Figure 2a) or diagnosed with clinically suspected (Figure 2b) tuberculosis. a**. Culture-confirmed Pulmonary Tuberculosis (N = 60). **b**. Clinically Suspected Tuberculosis (N = 92). Any participant who were LAM + but not diagnosed with culture-confirmed or clinically suspected tuberculosis are not depicted in these figures.

Among the 24 sputum AFB positive participants, 6 (25%) were urine LAM positive and 18 (75%) were urine LAM negative. Among the 60 participants with culture-confirmed pulmonary tuberculosis, urine LAM testing identified 12 (20%) participants more than sputum AFB, and sputum AFB testing identified 6 (10%) participants more than urine LAM (Figure 2a). Only 5 of 60 (8%) sputum culture-positive participants were identified by both sputum AFB and urine LAM testing.

Among the 92 participants with clinically suspected tuberculosis, urine LAM testing identified 17 (18%) participants more than sputum AFB, and sputum AFB testing identified 11 (12%) participants more than urine LAM (Figure 2b). Only 6 of 92 (7%) participants with clinically suspected tuberculosis were identified by both sputum AFB and urine LAM testing.

### Post-test Probability of Tuberculosis

**Figure 3:**
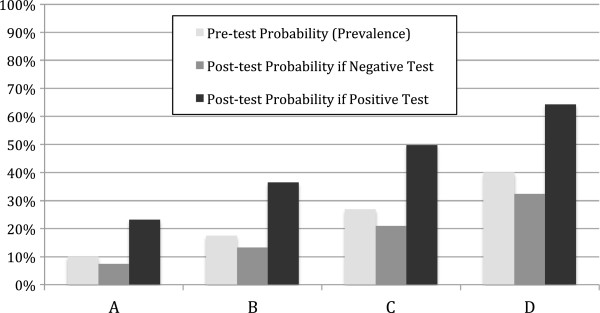
**Post-test probability of tuberculosis when using a combined screening strategy of sputum AFB and urine LAM testing for various pre-test probabilities.** Column A represents a cohort with 10% prevalence of tuberculosis; columns B and C represent the baseline prevalence of culture-positive pulmonary tuberculosis (17.5%) and clinically suspected tuberculosis (26.9%) that we observed in our cohort. Column D represents a cohort with a 40% prevalence of tuberculosis, which might occur among tuberculosis suspects in a highly endemic region.

A representation of post-test probability of tuberculosis when using a combined screening strategy of sputum AFB and urine LAM testing is shown in Figure 3. If the baseline prevalence of tuberculosis is 10% (column A), negative sputum AFB and urine LAM decreased the post-test probability to 7.4%, while either positive test increased the post-test probability to 23.1%. When assuming a prevalence of tuberculosis of 17.5% (column B), as observed for culture-positive pulmonary tuberculosis in our cohort, positive sputum AFB or urine LAM increased post-test probability to 36.4%. When assuming a prevalence of tuberculosis of 26.9% (column C), as observed for clinically suspected tuberculosis in our cohort, positive sputum AFB or urine LAM increased post-test probability to 49.8%. When assuming a higher pre-test probability of tuberculosis (40%; column D), negative sputum AFB and urine LAM decreased the post-test probability of tuberculosis to 32.4%, while either positive test increased post-test probability to 64.3%.

## Discussion

In this prospective, clinic-based study, the Determine™ TB LAM urine test had low sensitivity, or a high false negative rate, as a screening test for tuberculosis among newly-diagnosed HIV-infected adults in a tuberculosis-endemic region. However, the urine LAM test had comparable test sensitivity to sputum AFB, and, when combined with sputum AFB testing, added 20% to the true positive rate, though at the expense of identifying an additional 9.6% as false positives. Urine LAM testing was easily performed by trained nurses in an outpatient clinic, returned results within an hour of sample collection, and did not require electricity, reagents, specimen transport, or a highly-trained laboratory microscopist. Our data support a recent suggestion that the urine LAM test be used in conjunction with, and not in place of, current diagnostic tests for active tuberculosis [22].

Diagnostic accuracy of the Determine™ TB LAM test has been reported in laboratory- and hospital-based studies. In a retrospective, laboratory-based study using frozen urine samples, Lawn et al. found nearly identical test sensitivity (28.2%) for detecting culture-positive pulmonary tuberculosis, but a higher test specificity (98.6%), when using urine LAM as a screening test among HIV-infected adults in Cape Town [23]. Their higher test sensitivity may be a result of performing two sputum cultures for most (94.6%) participants, as well as performing urine LAM testing on frozen samples in a controlled laboratory-environment. In a study of HIV-infected TB suspects, 34% of whom were receiving antiretroviral therapy, a urine LAM testing had a sensitivity of 44.8% and specificity of 90.1% [24]. While the test specificity was similar to our findings, the higher test sensitivity was likely due to inclusion of patients with higher bacillary loads, as 34% of their participants had mycobacterium detected in blood cultures, as well as sicker hospitalized patients. We performed 2 urine LAM tests per sample, which improved sensitivity, but reduced overall test specificity. A second study of hospitalized HIV-infected patients reported test specificity of 90% when using the manufacturer’s threshold of positive versus negative, which was similar to our findings [25]. In contrast to these study, our results represent diagnostic accuracy when trained nurses use Determine™ TB LAM testing as a screening test in newly-diagnosed HIV-infected adults in a real-world outpatient clinical setting, where patients generally have less advanced disease compared to hospitalized patients.

Although the rapid urine LAM test was comparable to sputum AFB for detecting active tuberculosis, when combined the two tests improved detection of tuberculosis. In a retrospective study by Lawn et al., adding urine LAM testing to sputum AFB testing increased overall sensitivity from 28.2% to 43.5%, while having minimal impact on test specificity [23]. While the change in sensitivity was similar to our study results, we found a large decrease in test specificity, or a higher false negative rate. Other studies have found the laboratory-based ELISA urinary LAM test capable of detecting extra-pulmonary tuberculosis [26]. The addition of urine LAM to existing screening strategies would be relatively inexpensive, and the rapid urine LAM test has been reported to be cost-effective when used to diagnose HIV-infected adults with CD4 < 100/mm^3^ and symptoms of tuberculosis [27]. In our cohort, urine LAM detected one-third of participants with extrapulmonary TB. The price for one LAM test, including a disposable pipette, was US $3.05, or $6.10 for our two-test screening strategy.

Utilizing rapid urine testing for active tuberculosis is appealing for several reasons. First, as we demonstrated in this study, the Determine™ TB LAM urine test is a true clinic-based, point-of-care test that can be used by trained nurses in peripheral clinics or remote settings without electricity or reliance on laboratory infrastructure. Second, participants were 3 times more likely to produce a urine sample than a sputum sample, which has been similarly reported in another study [23]. This difference could have additional clinical benefit for urine LAM testing, but would need to be evaluated in an operational study [28]. Third, obtaining urine samples from tuberculosis-infected patients carries a lower risk of transmission to health care workers than sputum specimens containing live, active bacilli. Finally, urine LAM has the potential for detection of extrapulmonary tuberculosis and may be a valuable biomarker of tuberculosis resolution during anti-tubercular therapy.

Our study had several limitations and strengths. Trained nurses performed specimen collection and urine LAM testing in an outpatient clinic to assess diagnostic accuracy when used at the clinical, not hospital, point-of-care in a real-world setting. While trained nurses may not interpret test results as accurately as a certified laboratory technicians, these results are more consistent with the intended use of the rapid, point-of-care test. If nurses had interpreted the appearance of a weak, faint test line as positive, this could have resulted in reduced test specificity. We obtained one sputum sample for the gold standard test of mycobacterial culture, while some studies of diagnostic accuracy included two or three specimens [23,24]. Sputum culture is an imperfect gold standard test and non-differential misclassification could lead to reduced diagnostic accuracy. We did not perform Xpert MTB/RIF testing, obtain mycobacterial blood cultures, perform testing on those unwilling to share their HIV status, score the positive urine LAM tests, obtain user performance evaluation data, obtain data on clinical reasons for initiating TB treatment, evaluate causes of extrapulmonary tuberculosis, measure inter-observer variability, or measure the bacillary burden of tuberculosis. Finally, we evaluated urine LAM as an outpatient screening test among HIV-infected adults with and without tuberculosis symptoms in a tuberculosis-endemic region, and these results may not be generalizable to other populations or areas with low tuberculosis rates.

## Conclusion

In conclusion, although a generalized role for urine LAM testing may be limited by poor test sensitivity, the rapid urine LAM test added important diagnostic case detection when combined with sputum AFB as a screening test for tuberculosis among newly-diagnosed HIV-infected adults in a tuberculosis-endemic region. In the absence of a highly accurate clinic-based, point-of-care test for active tuberculosis, clinic-based urine LAM testing offers a rapid, inexpensive option with diagnostic yield that is similar, but complementary, to sputum smear microscopy. Urine LAM testing may be beneficial in settings with limited capacity for sputum smear microscopy or when a timely diagnosis of TB is paramount, but would greatly benefit by yielding a higher test sensitivity. Since urine LAM testing has several appealing point-of-care properties, clinical studies are warranted to determine whether urine LAM testing influences patient outcomes.

## Competing interests

We declare that we have no conflicts of interest.

## Authors’ contributions

PKD, EL, and IVB designed the study. PKD, SMC, JG, DR, and IVB collected and assembled data. EL and SMC performed statistical analysis. PKD wrote the report with input from EL, SMC, JG, DR, JNK, RPW, KAF, and IVB. All authors approved the final version of the article.

## Pre-publication history

The pre-publication history for this paper can be accessed here:

http://www.biomedcentral.com/1471-2334/14/110/prepub
